# Shared and independent roles of CGRP and PACAP in migraine pathophysiology

**DOI:** 10.1186/s10194-023-01569-2

**Published:** 2023-04-03

**Authors:** Adisa Kuburas, Andrew F. Russo

**Affiliations:** 1 Department of Molecular Physiology and Biophysics and Department of Neurology, University of Iowa, Iowa City, IA 52242 USA; 2 Veterans Affairs Medical Center, Iowa City, IA 52246 USA

**Keywords:** CGRP, PACAP, Migraine, Intracellular signaling, Receptors

## Abstract

The neuropeptides calcitonin gene-related peptide (CGRP) and pituitary adenylate cyclase-activating polypeptide (PACAP) have emerged as mediators of migraine pathogenesis. Both are vasodilatory peptides that can cause migraine-like attacks when infused into people and migraine-like symptoms when injected into rodents. In this narrative review, we compare the similarities and differences between the peptides in both their clinical and preclinical migraine actions. A notable clinical difference is that PACAP, but not CGRP, causes premonitory-like symptoms in patients. Both peptides are found in distinct, but overlapping areas relevant to migraine, most notably with the prevalence of CGRP in trigeminal ganglia and PACAP in sphenopalatine ganglia. In rodents, the two peptides share activities, including vasodilation, neurogenic inflammation, and nociception. Most strikingly, CGRP and PACAP cause similar migraine-like symptoms in rodents that are manifested as light aversion and tactile allodynia. Yet, the peptides appear to act by independent mechanisms possibly by distinct intracellular signaling pathways. The complexity of these signaling pathways is magnified by the existence of multiple CGRP and PACAP receptors that may contribute to migraine pathogenesis. Based on these differences, we suggest PACAP and its receptors provide a rich set of targets to complement and augment the current CGRP-based migraine therapeutics.

## Background

Migraine is one of the most disabling neurological disorders in the world [1]. It affects over one billion people with 3:1 prevalence in women. Migraine is a headache lasting 4–72 h with characteristics that often include unilateral pulsating pain of moderate to severe intensity that is aggravated by routine physical activity and is associated with vomiting or nausea and/or photophobia and phonophobia [2]. While the mechanisms of migraine are still poorly understood, insights from clinical and preclinical studies over the past three decades have focused attention on two neuropeptides: calcitonin gene-related peptide (CGRP) and pituitary adenylate cyclase-activating polypeptide (PACAP).

The importance of CGRP in migraine was first realized in reports that CGRP is upregulated during migraine attacks and between attacks in chronic migraine patients [3–5], although this is not seen in all studies [6–8]. Even more striking was the finding that infusion of CGRP can induce migraine-like attacks in migraine patients, as described below. The importance of CGRP has been fully established over the past 5 years with the efficacy of eight FDA approved CGRP-based therapeutics [9–13]. These drugs include monoclonal antibodies against CGRP or its receptor and small molecule receptor antagonists that are effective for prevention and treatment of migraine. However, in general only about 40–60% of migraine patients are significantly helped by these agents [12, 14, 15], which suggests involvement of other factors beyond CGRP in migraine pathophysiology, such as PACAP [16]. In this context, patients who do not respond well to CGRP-based drugs might respond to drugs that target PACAP and similarly, a combinatorial approach targeting both CGRP and PACAP might improve treatment efficacies.

Like CGRP, PACAP has been linked to migraine pathogenesis [17–19]. The PACAP gene encodes two isoforms containing either 27 or 38 amino acids with PACAP-38 being the more prevalent, representing 90% of PACAP forms in mammalian tissues [20, 21]. Unless otherwise indicated, we will refer to both isoforms simply as PACAP. As with CGRP, elevated plasma PACAP levels during migraine have been reported [19, 22], but not consistently observed [23, 24], and infusion of either the PACAP-38 or PACAP-27 isoforms caused migraine in people, as described below [18, 25].

The goal of this narrative review is to briefly compare and contrast the actions of CGRP and PACAP in migraine patients and rodent migraine models. For more extensive reviews on CGRP and PACAP, the reader is referred to a number of excellent reviews [23, 26–33].

## Infusion of CGRP and PACAP in patients

A key similarity of CGRP and PACAP is their ability to induce migraine-like headaches when infused into migraine patients (Table 1). Intravenous infusion of CGRP caused a delayed migraine-like headache in about 63% of migraine patients (50–77%), but only a mild immediate headache in control subjects [34–40]. Similarily, intravenous infusion of PACAP-38 caused a delayed migraine-like headache in about 68% of migraine patients (58–73%), but only rarely in control subjects [17, 18, 38, 41]. This was also seen with the shorter PACAP isoform, PACAP-27 [25]. In addition, both CGRP and PACAP-induced attacks in migraine patients were effectively treated by sumatriptan [35, 42]. However, sumatriptan did not block CGRP-induced headaches in control subjects [43], which emphasizes the importance of doing infusion studies in migraine patients.

**Table 1:** Comparison of migraine frequencies after infusion of CGRP, PACAP, and other triggers in humans

Trigger^a^	Migraine Frequency^b^	References
CGRP	63% (50–77%)	50%^c^, [34] 57%, [36] 75%, [35] 63%, [37, 38]^d^ 77%, [39] 56%, [40]
PACAP-38	68% (58–73%)	58%, [18] 73%, [17] 72%, [38, 41]^d^
PACAP-27	55%	[25]
VIP (20 min infusion)	9% (0–18%)	0%, [44] 18%, [17]
VIP (2 h infusion)	71%	[45]
Pramlintide (amylin analog)	41%	[40]
Adrenomedullin	55%	[46]
Glyceryl trinitrate (GTN)	70% (67–80%)	67%, [47] 80%, [48] 50%, [49] 75%, [50] 77%^e^, [51]
Sildenafil (PDE5^f^ inhibitor)	83%	[52]
Dipyridamole (PDE5^f^ inhibitor)	50%	[53]
Cilostazol (PDE3^f^ inhibitor)	86%	[54]
Levcromakalim (K_ATP_ channel opener)	91% (82–100%)	100% [55], 82%, [56]
Histamine	70%	[57]
Prostaglandin E_2_	58%	[58]
Prostaglandin I_2_	50%	[59]

^a^Administrations were by intravenous infusion (~ 20 min), except for sublingual GTN in two studies [47, 51] and oral delivery of sildenafil and cilostazol

^b^The average frequency and range of all migraine attacks are combined from migraine patients with and without aura. Data do not include familial hemiplegic migraine, traumatic brain injury, or control subjects

^c^While originally reported as 33%, this was subsequently revised to 50% [36]

^d^Further descriptions of the same patients as in [38]

^e^Average frequency from both the migraine without and with aura cohorts

^f^PDE = phosphodiesterase

The frequencies of CGRP and PACAP induced attacks were comparable to those observed with other migraine triggers (Table 1). These triggers include other members of the CGRP and PACAP families, the nitric oxide donor glyceryl trinitrate (GTN), phosphodiesterase inhibitors that elevate cAMP and cGMP levels, an activator of ATP-sensitive potassium (K_ATP_) channels, and inflammatory agents (histamine, prostaglandins).

With respect to identifying the relevant receptors for CGRP and PACAP involved in migraine, it is informative that other members of the CGRP and PACAP peptide families can induce migraine. Two CGRP-related peptides, adrenomedullin and a synthetic analog of amylin (pramlintide), triggered migraine-like attacks (Table 1) [40, 46]. As discussed below, CGRP, amylin, and adrenomedullin act via a family of related G protein-coupled receptors (GPCRs) and in particular, CGRP binds the amylin 1 (AMY_1_) receptor with equal affinity as its canonical receptor [26]. In the case of PACAP, the family member vasoactive intestinal peptide (VIP) caused migraine-like headaches comparable to PACAP, but only after prolonged infusion to mimic the longer lasting vascular actions of PACAP [60]. The shared ability of PACAP and VIP is important since while the PACAP1 (PAC_1_) receptor is preferentially activated by PACAP, the two peptides are equally active at the VIP-PACAP (VPAC) receptors VPAC_1_ and VPAC_2_, as discussed below.

**Fig. 1:**
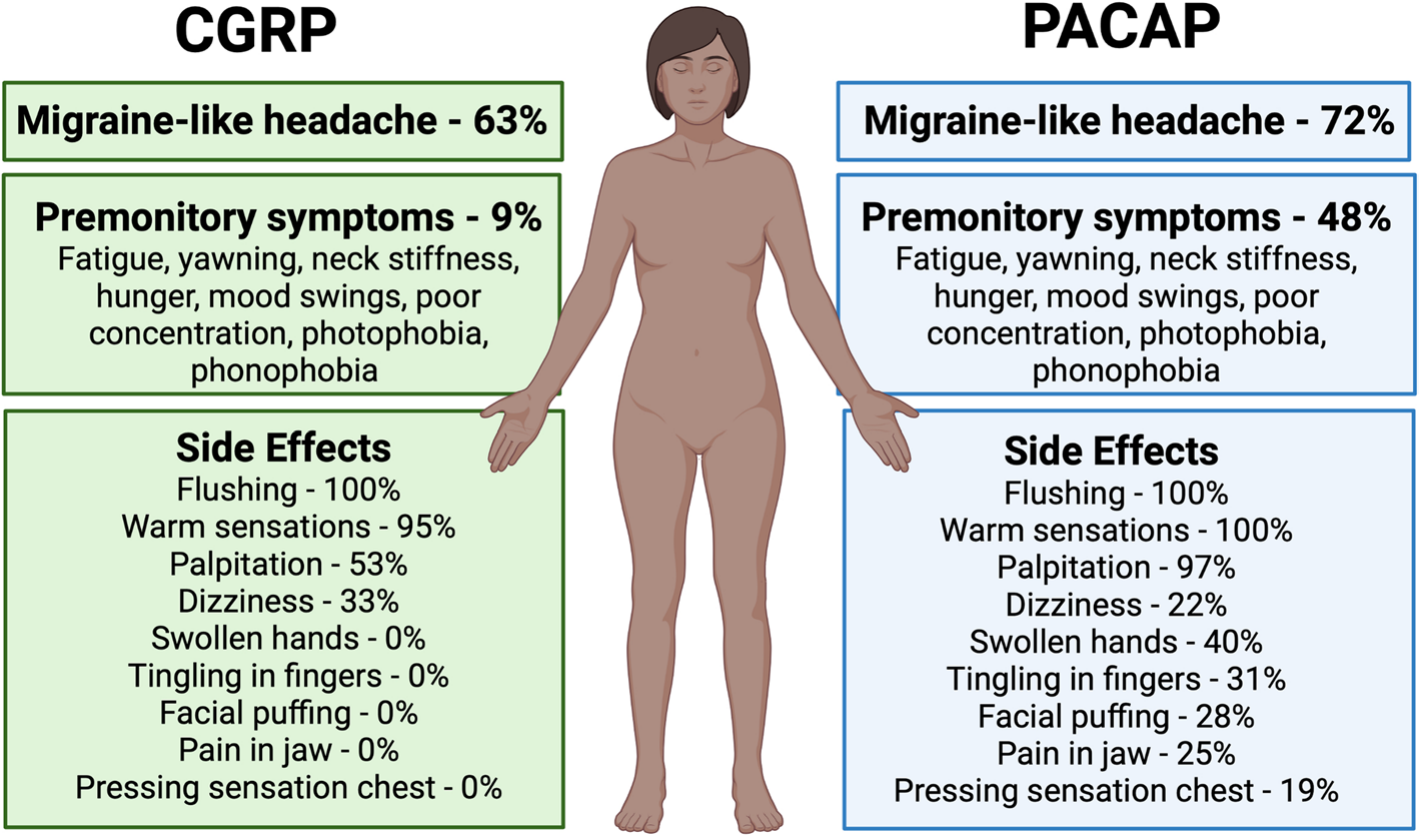
Clinical symptoms caused by CGRP and PACAP infusions. Both CGRP and PACAP cause migraine-like headache in about 2/3 of migraine patients. PACAP causes more premonitory symptoms and side effects than CGRP. Data are only from studies that included premonitory symptoms [37, 38, 41]. For a comprehensive listing of CGRP and PACAP infusion studies and migraine frequencies, see Table 1. Created with BioRender.com

In migraine patients, CGRP also induced non-headache symptoms characteristic of migraine, including photophobia, phonophobia, and nausea. In addition to headache, cranial vascular changes were observed with dilation of the middle cerebral artery (MCA) and middle meningeal artery (MMA) [35, 61, 62]. Like CGRP, PACAP also induced photophobia and other non-headache symptoms. As with CGRP, there were cranial vascular changes. PACAP-induced headache was associated with prolonged dilation of the MMA but not the MCA [17, 63]. Both CGRP and PACAP caused side effects likely due to systemic vasodilation (flushing, warm sensation, palpitation, dizziness), although PACAP caused additional effects not seen with CGRP-infusion [37, 38, 41] (Fig. 1).

A difference between CGRP and PACAP was revealed when patients were asked if they developed premonitory symptoms after peptide infusion (Fig. 1). Premonitory symptoms occur prior to the headache in most migraine patients [64, 65]. Premonitory symptoms most commonly observed include fatigue, yawning, neck stiffness, hunger or food cravings, mood swings, poor concentration, and sometimes photophobia and phonophobia, which also occur during the headache phase. After PACAP infusion, a delayed migraine-like headache was reported by 23 of 32 patients (72%) and 11 of those 23 (48%) reported one or more premonitory symptoms prior to the headache [38]. In contrast, after CGRP infusion, while migraine was reported by 25 of 40 patients (63%), only 2 of those 25 (9%) reported premonitory symptoms prior to the headache. This difference in premonitory symptoms between CGRP and PACAP may reflect PACAP's ability, albeit limited, to enter the central nervous system (CNS) [66]. Within the CNS, the hypothalamus has been strongly associated with the premonitory phase by imaging studies [67, 68] and other criteria [69]. Importantly, the hypothalamus has abundant PACAP receptors [70]. However, caution must be exercised in interpreting these results due to several caveats, most notably the lack of placebo and non-migraine control groups [38]. These caveats are particularly important since CGRP and PACAP induced premonitory symptoms to the same extent in patients who did not develop a migraine attack as those who did, which raises the prospect that patients may have been exhibiting peptide responses that were not necessarily premonitory of migraine. Hence, it might be safer to refer to the symptoms as premonitory-like. Nonetheless, it is clear that both CGRP and PACAP can induce a delayed migraine-like headache, and that PACAP can also initiate premonitory-like symptoms.

To understand the mechanism of CGRP and PACAP induced headaches, Ashina and colleagues tested whether CGRP and PACAP share K_ATP_ channels as a downstream cellular target. K_ATP_ channels are ATP regulated potassium channels located in trigeminovascular neurons and vessels. The rationale of this idea was based on studies showing that the K_ATP_ channel opener levcromakalim was a potent inducer of migraine in patients (Table 1) [55, 56], and that both CGRP and PACAP elevate cAMP levels, which in vascular smooth muscle would activate the channels, leading to vasodilation associated with headache [71]. Yet neither CGRP nor PACAP actions were blocked by treatment with an inhibitor of K_ATP_ channels, glibenclamide [72, 73]. However, the lack of efficacy of glibenclamide must be tempered by the caveats that the studies were not done in migraine patients and glibenclamide only delayed and did not prevent levcromakalim-induced headaches [74]. Furthermore, preclinical allodynia studies described below showed that glibenclamide inhibits CGRP, but not PACAP actions in mice. Further studies with glibenclamide and other antagonists in migraine patients are needed to help resolve this discrepancy.

## Migraine relevant sites of CGRP and PACAP and their receptors

**Fig. 2:**
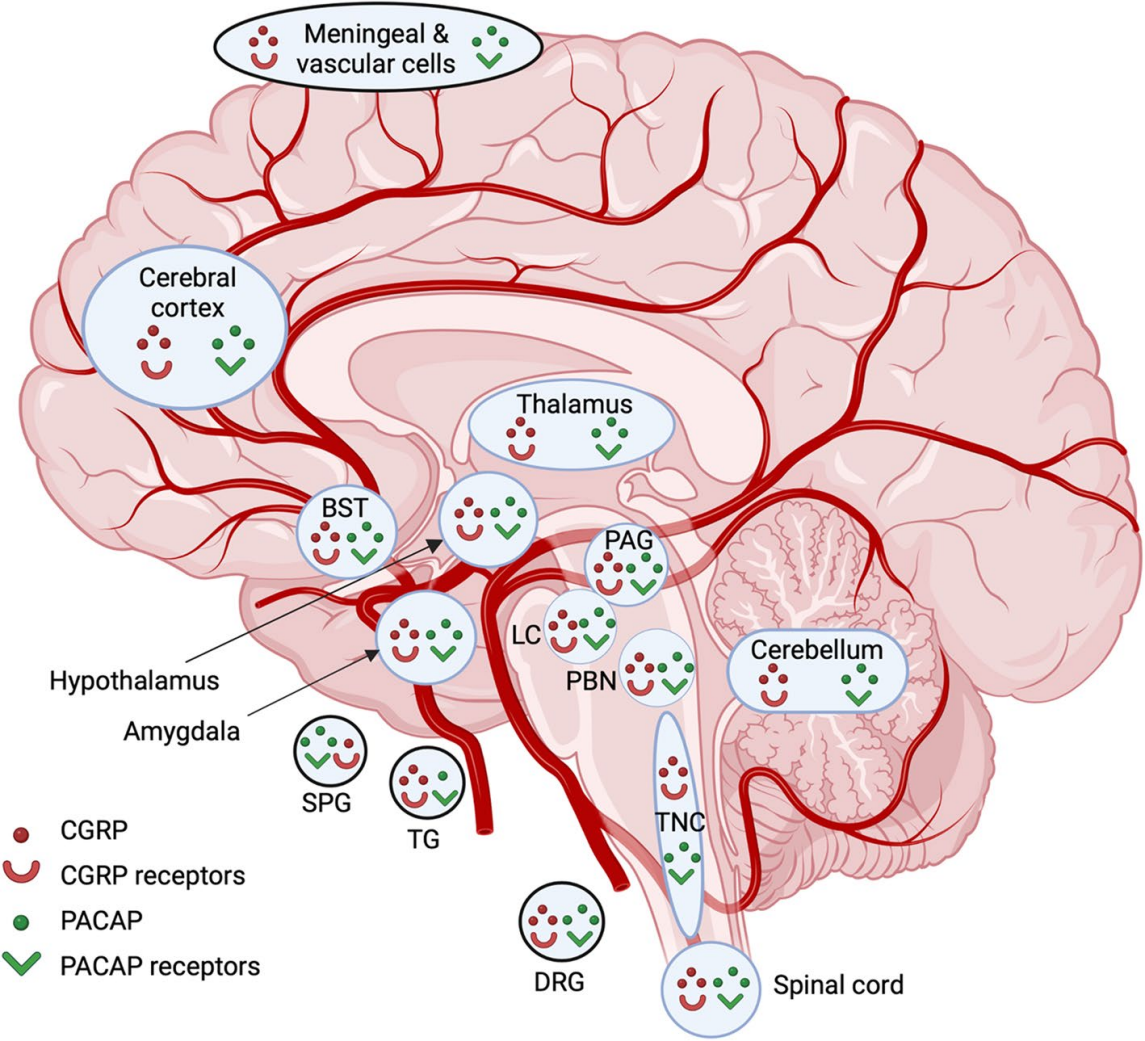
Sites of CGRP, PACAP, and their receptors in the CNS and cranial structures. CGRP, PACAP and their receptors are present in meningeal and vascular cells [77–80], hypothalamus [75, 76], thalamus [75, 76], amygdala [76, 81–83], cerebellum [75, 76], cerebral cortex [75, 76], sphenopalantine ganglia (SPG) [84–87], bed nucleus of stria terminalis (BST) [81, 83, 88, 89], periaqueductal gray (PAG) [90–92], locus coeruleus (LC) [75, 76], trigeminal nucleus caudalis (TNC) [93–95], parabrachial nucleus (PBN) [81, 83, 96, 97], trigeminal ganglia (TG) [98–100], dorsal root ganglia (DRG) [98, 101–103], and spinal cord [91, 93, 104]. Peripheral cranial structures are indicated with a black circle. For peptides, location within a region indicates presence in cell bodies and/or fibers. Relative abundance or cellular resolution of the two peptides or their receptors have generally not been directly compared, with the exception of the TG and SPG, where the relative abundances of CGRP over PACAP in the TG and PACAP over CGRP in the SPG are indicated. For receptors, location in a region is a collective assessment of CGRP receptors (canonical CGRP, AMY_1_) and PACAP receptors (PAC_1_, VPAC_1_, VPAC_2_, MRGB2/B3/X2). Created with BioRender.com

Based on the shared ability of exogenous CGRP and PACAP to cause migraine, a pertinent question is where are endogenous sites of CGRP and PACAP expression and action in the central and peripheral nervous systems? Both peptides and their receptors are found in multiple areas relevant to migraine, ranging from the hypothalamus to the trigeminal ganglia (Fig. 2). These sites largely overlap, but there are differences and few studies have looked at cellular co-expression other than in the trigeminal and sphenopalatine ganglia [75, 76].

In the peripheral nervous system, CGRP is predominantly expressed in sensory neurons of the dorsal root and trigeminal ganglia, although it is also found in motor neurons and is abundant within the enteric nervous system [26]. The distribution of CGRP appears to be largely similar across species (reviewed in [26]). In the mouse, rat and human trigeminovascular system, CGRP is primarily found in the perivascular afferents innervating cranial arteries [105, 106]. Within rat and human trigeminal ganglia, PACAP and CGRP are found in neurons, and PACAP receptors are found on both neurons and satellite glia [98, 107–109]. While co-localized with CGRP, PACAP is found in far fewer neurons [110]. Although, PACAP receptors PAC_1_ and VPAC_1_ are found in rat and human satellite glia [111], their functions are not known. CGRP receptors are also found on subsets of trigeminal ganglia neurons and satellite glia in rats, mice and humans, where they may contribute to peripheral sensitization in migraine [26, 106, 112].

In contrast to CGRP, in the periphery of the cranium, PACAP is mainly expressed in parasympathetic neurons with a much smaller trigeminal distribution than CGRP in rats and humans [99, 110]. The predominant site of PACAP expression in rats and humans is the extracranial parasympathetic sphenopalatine ganglion, which also contains PACAP receptors [84, 85, 113]. Stimulation of the sphenopalatine ganglia likely contributes to autonomic symptoms of migraine since it can increase cerebral blood flow, intracranial and extracranial vasodilation, and dural plasma protein extravasation in humans [114]. Within the sphenopalatine ganglia, CGRP containing fibers from the trigeminal ganglia were found in both rat and human [86, 87]. CGRP was also found in neural cell bodies but only in rats, not humans [86]. Interestingly, PACAP can induce release of CGRP from rat trigeminal neurons [107] and stimulation of the rat superior salivatory nucleus can activate neuronal trigeminovascular actions and cranial autonomic symptoms [115, 116]. These results all suggest the possibility of cross-talk between the sphenopalatine and trigeminal systems.

Within the CNS, both peptides and their receptors are found in migraine-relevant regions across species, especially the hypothalamus (Fig. 2) [26, 70]. CGRP receptors have been identified throughout the CNS and are particularly abundant in the human cerebellum [117]. PACAP is found in the spinal cord and second order neurons of the trigeminal nucleus caudalis (TNC) of rodents and humans [93, 118, 119]. Similar to CGRP, PACAP binds to a variety of sites throughout the CNS, including the hypothalamus, thalamus, various areas throughout the brainstem, and the dorsal horn of the spinal cord across species [120–122]. In particular, the PAC_1_ receptor is expressed throughout the brain, including the neocortex, limbic system, and brainstem [123]. Like CGRP [26], PACAP has been linked to anxiety-like behavior [124, 125]. PACAP and PAC_1_ knockout mice have decreased anxiety-like behavior. Both knockouts show a variety of neurobehavioral phenotypes including increased hyperactivity, decreased depression-like behavior, and aberrant social interaction [124, 125]. Studies have identified a genetic association with PACAP and the PAC_1_ receptor with post-traumatic stress disorder in humans and shown that alterations in the PACAP/PAC_1_ pathway are involved in stress responses in rodents [126]. In addition, chronic stress increased PACAP expression within the rat bed nucleus of the stria terminalis [127]. These findings document a role for PACAP in stress and anxiety, which are both associated with migraine [128]. Hence, the locations of CGRP and PACAP peptides and their receptors are overlapping and well-positioned to contribute to peripheral and central actions in migraine.

## CGRP and PACAP migraine-like functions

### CGRP and PACAP roles in vasodilation, neurogenic inflammation, and nociception

Both CGRP and PACAP are multifunctional peptides with many roles in the nervous, cardiovascular, respiratory, gastrointestinal, and reproductive systems [26, 123, 129]. We will briefly focus on three processes that are associated with migraine: vasodilation, neurogenic inflammation, and nociception. While the role of vasodilation and neurogenic inflammation in migraine remains a debated topic, and neurogenic inflammation has not led to a successful therapeutic, it does seem likely that the vasculature and local inflammatory signals contribute to peripheral sensitization and hence to migraine [26, 130].

Both CGRP and PACAP are well-characterized vasodilatory peptides [131], and as mentioned above both can act on cranial vessels. It is intriguing that the two peptides, along with another commonly used migraine trigger, GTN (a nitric oxide donor), are all vasodilators [132]. In addition to their contributions via vasodilation in neurogenic inflammation in rats [133, 134], CGRP and PACAP cause mast cell degranulation and release of inflammatory compounds. These CGRP actions are well-documented in the rat dura [80]. PACAP-38 was reported to induce dural mast cell degranulation in rats and was significantly more potent than VIP and PACAP-27 [135, 136]. Like CGRP, PACAP is upregulated following inflammation in sensory neurons [137]. However, the complexity of PACAP actions is highlighted by the fact that in contrast to the dura, PACAP inhibits neurogenic inflammation in rodent skin [138–141]. Nonetheless, within the meninges, it seems likely both PACAP and CGRP can contribute to neurogenic inflammation.

With respect to nociception, the story is even more complex. While CGRP is recognized as a nociceptive peptide [26], PACAP appears to have both antinociceptive and nociceptive functions. In the periphery, PACAP was reported to be antinociceptive [138–141]. In contrast, PACAP in the CNS appears to be nociceptive based on studies with PACAP knockout mice suggesting a possible role in central sensitization [141]. Similarly, injection of PACAP into the hypothalamic paraventricular nucleus increased TNC activity in rats, which could be inhibited by a PAC_1_ receptor antagonist [142] and intrathecal injection of PACAP has been shown to induce hyperalgesia in mice [122]. PACAP also causes a delayed activation and sensitization of central trigeminovascular neurons. Specifically, the central PAC_1_ receptors have been implicated in pro-nociceptive transmission. A centrally, but not peripherally, administered PAC_1_ receptor antagonist was able to inhibit dural nociceptive-evoked action potentials in central trigeminovascular neurons in rats, suggesting that the central PAC_1_ receptor is involved in PACAP-induced migraine [143].

### Light aversion induced by CGRP and PACAP in mice

A shared activity of CGRP and PACAP is their ability to induce similar light aversive phenotypes in mice [144]. The light aversion assay serves as a surrogate for human photophobia [145, 146]. Central (intracerebroventricular, thalamic, and cerebellar) and peripheral (intraperitoneal) injection of CGRP induced light aversion in wildtype mice [144, 147–150]. CGRP-induced light aversion was accompanied by increased resting only in the dark zone, and a lack of light-independent anxiety in an open field assay [147, 151–153]. Likewise, intraperitoneal injection of PACAP caused light aversion coupled with increased resting in the dark and no anxiety in the open field [144]. These findings are consistent with a pioneering study by Helyes and colleagues who reported that peripheral injection of GTN and PACAP induced light aversive behavior in wildtype mice, but not in PACAP knockout mice [154]. It should be noted that compared to PACAP-38, injection of PACAP-27 caused only transient light aversion [144]. A pharmacokinetic explanation cannot be ruled out since the relative stability of the two PACAP isoforms is not clear [21]. However, it is possible that PACAP-38, but not PACAP-27, acts by mast cell degranulation, as shown for dilation of the MMA in rats [155]. In fact, in rats only PACAP-38 can degranulate mast cells and acts via the Mas-related GPCR B3 (MrgB3) receptor [156]. Studies exploring the role of the MrgB3 homologs in mice (MrgB2) and humans (MRGX2) may give insights to how PACAP-38 evokes symptoms of migraine.

Despite the similarities, CGRP and PACAP act independently in the light aversion assay. This was shown by the fact that CGRP and PACAP responses could not be blocked by monoclonal antibodies directed against the other peptide [144]. Hence, PACAP-induced responses could be blocked with a monoclonal anti-PACAP antibody, but not by an anti-CGRP antibody. Conversely, CGRP-induced responses could be blocked by an anti-CGRP antibody, but not by an anti-PACAP antibody. This result suggests that CGRP and PACAP do not act by sequential or dependent pathways. The possibility of dependent actions had been raised by the similar properties of CGRP and PACAP [131], co-expression in rat trigeminal ganglia neurons [99], and PACAP-38 causing CGRP release in the rat TNC (although not from the dura or ganglia) [107]. Contrary to the latter observation in rats, a clinical study did not detect increased CGRP levels after PACAP-38 infusion [24]. Furthermore, GTN increased the number of PACAP-responsive neurons in mouse trigeminal ganglia by a mechanism independent of CGRP [157]. In contrast, the parallel increase in CGRP-responsive neurons required CGRP. These data all suggest that PACAP and CGRP can act by distinct pathways that converge downstream of the receptors to cause migraine-like symptoms.

Further support for CGRP and PACAP acting by different pathways to cause light aversion is that PACAP was effective in only a subpopulation of CD-1 mice and their offspring, which was not seen with CGRP [144]. The CD-1 strain is a genetically diverse outbred strain of mice, which raised the possibility of genetic differences between the responder and nonresponder populations. An RNA-seq analysis of trigeminal ganglia gene expression between the two populations revealed a number of candidate genes, including pituitary hormones, receptors, and ion channels that are potential biomarkers and therapeutic targets. Whether these genes will provide clues for identifying human responder/nonresponder populations remains to be seen but this finding of heterogeneity reflects an advantage of using genetically diverse mice that may better model the variability observed in humans [158].

### Allodynia induced by CGRP and PACAP in rodents

Subcutaneous injection of CGRP in the periorbital area in mice caused dose and time dependent mechanical allodynia [159]. This CGRP-induced periorbital allodynia was abolished by pretreatment with a CGRP receptor antagonist (olcegepant) or a monoclonal anti-CGRP antibody [159]. Similar allodynia was also induced by intraperitoneal and intrathecal injections of CGRP in mice [160, 161] and intraganglionic injections of CGRP into rat trigeminal ganglia [162]. Subcutaneous injection of PACAP in the periorbital area also caused dose and time dependent mechanical allodynia and was blocked by pretreatment with a PACAP receptor antagonist, PACAP6-38 [159]. Similarly, subcutaneous injection of PACAP induced plantar and periorbital hypersensitivity in wildtype mice [163].

Consistent with the light aversion findings, CGRP and PACAP-induced allodynia appears to act via independent pathways as reported by Christensen and colleagues. They observed PACAP responses in wildtype mice pretreated with anti-CGRP antibody, as well as in Ramp1 knockout mice lacking CGRP receptors [163]. For comparison, allodynic responses to GTN treatments were blocked by anti-CGRP antibodies in wildtype mice and not seen in the Ramp1 knockout mice [164]. This indicates that PACAP acts independently of CGRP signaling. Separate pathways were also suggested by pretreatment with the K_ATP_ channel inhibitor glibenclamide. Glibenclamide was able to block GTN-induced allodynia in mice, which involves CGRP [164, 165], but only partially attenuated PACAP-induced hypersensitivity, indicating that PACAP does not fully depend on this channel [163]. A follow up study showed that pretreatment with anti-PACAP antibody blocked PACAP-induced plantar hypersensitivity but was not able to block hypersensitivity caused by GTN or the K_ATP_ channel opener levcromakalin [166]. However, a caveat of these comparisons is that they did not directly test glibenclamide or PACAP antibodies against CGRP, but rather against GTN, which acts via CGRP, at least in rodents. Glibenclamide was also able to attenuate cephalic allodynia in spontaneous trigeminal allodynic rats and inhibited release of CGRP from dura mater and trigeminal ganglion [165]. Yet, translation of these rodent studies to migraine patients remains to be established, since as mentioned earlier, glibenclamide was unable to block CGRP or PACAP-induced headache in control subjects [72, 73]. While these findings suggest that CGRP and GTN act by pathways not shared with PACAP, other data link PACAP to nitric oxide pathways. Peripheral injection of GTN increased PACAP within the rat TNC [119], and increased the number of PACAP-responsive neurons in mouse trigeminal ganglia [157]. Also, GTN induced more vasodilation and neuronal activation in trigeminal ganglia and the TNC in wildtype mice compared to PACAP knockout mice [154]. Taken together, while there seems to be some cross-talk between CGRP, PACAP and nitric oxide in the trigeminovascular system, it appears that PACAP and CGRP can act by independent pathways to cause tactile and light sensitivities.

## Signaling by multiple CGRP and PACAP receptors

**Fig. 3:**
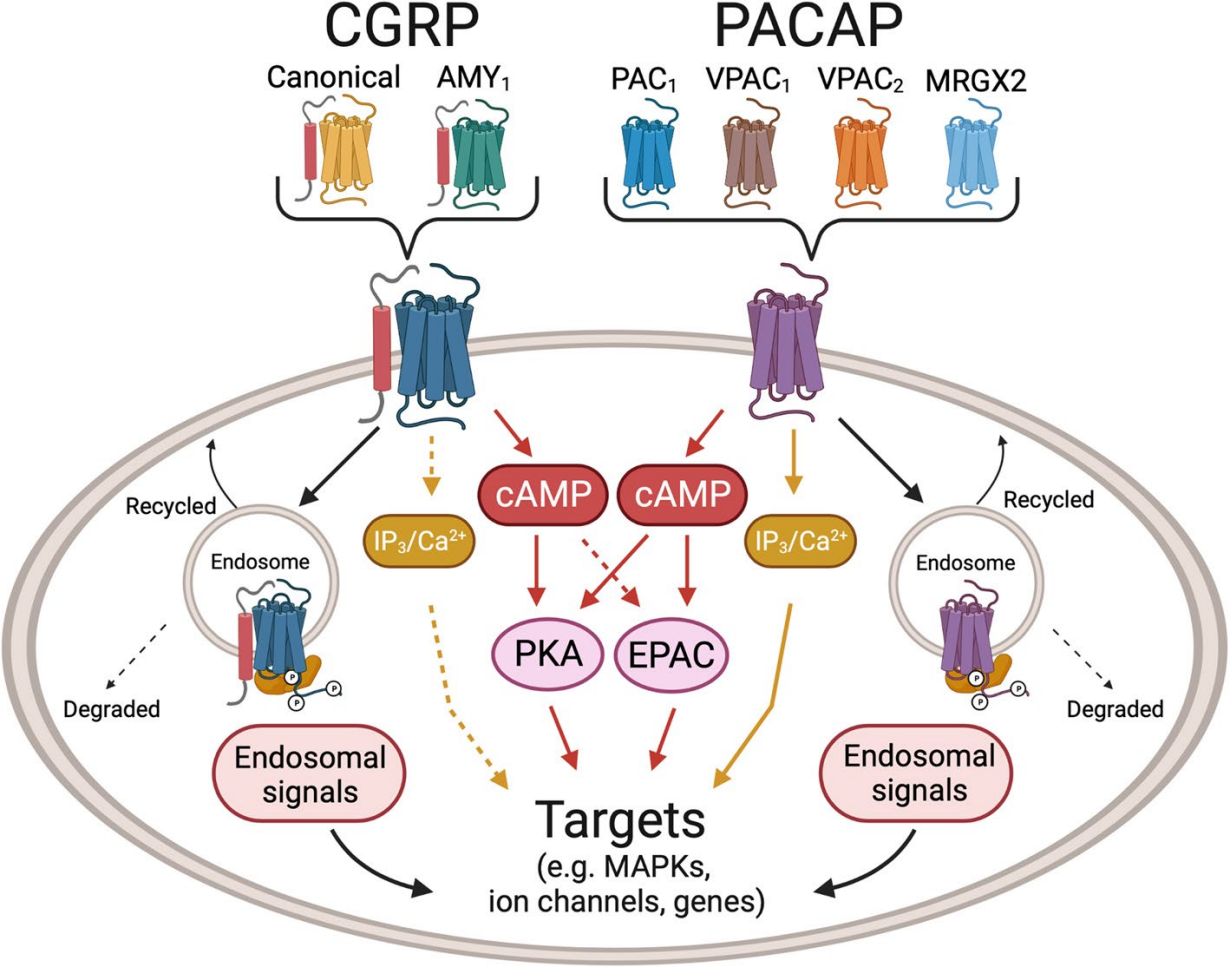
Schematic of CGRP and PACAP signaling pathways. CGRP and PACAP can act via multiple receptors, as indicated. For simplicity, signaling pathways from generic receptors in a generic cell type are illustrated. In general, activation of both CGRP and PACAP receptors increase cAMP levels, which leads to protein kinase A (PKA) activation and EPAC1/2 activation. EPAC1/2 activation by PACAP is well-established, although activation by CGRP is less clear (dotted line). Canonical CGRP receptor, but not AMY_1_ CGRP receptor, and the PACAP receptor PAC_1_ can generate endosomal signals following β-arrestin-mediated receptor internalization. Activation of additional G protein pathways that elevate IP_3_ and calcium have been reported for PACAP and to a lesser extent CGRP (dotted lines). These pathways activate multiple downstream targets, including MAP kinases (MAPKs), ion channels, and genes, depending on the cell type. Created with BioRender.com

CGRP and PACAP receptors are both Gs-coupled and activate cAMP-dependent pathways [26, 123, 167–170] (Fig. 3). In addition, both peptides have been shown to activate MAP kinase pathways and are reported to couple to Gq, which signals via calcium pathways involving phospholipase C and inositol 1,4,5-triphosphate (IP_3_) activity [169, 171, 172]. However, conflicting results have been reported for the direct measurement of coupling of CGRP receptors to Gq [173, 174]. Furthermore, identification of Gq coupling has been mostly inferred from CGRP mediated calcium mobilization and IP_3_ signaling [175, 176]. Interestingly, in one report of CGRP signaling via Gq, the authors suggested that these cells did not have a cAMP response to CGRP [177]. Perhaps the absence of Gs and/or the high expression of Gq allows preferential Gq activation. In contrast, PACAP receptors have more robust evidence for Gq-mediated signaling [178], although direct comparisons between PACAP and CGRP receptors can be difficult to make due to differences in model systems. In a series of studies using the same transfected receptor model, CGRP mediated stimulation of IP accumulation (measured as IP_1,_ a breakdown product of IP_3_) was > 200 fold less potent relative to cAMP [179, 180]. Whereas, PACAP- mediated stimulation of IP was only approximately 4–tenfold less potent relative to cAMP [181]. Hence, IP signaling appears to be more robust for PACAP receptors than CGRP receptors. Overall, it seems that the CGRP receptor can couple to Gq, but the coupling may not be as robust as Gs coupling. Whereas PACAP receptors appear to effectively couple both Gq and Gs.

Thus, CGRP and PACAP receptors have the potential to activate similar intracellular signaling pathways that could lead to a host of cellular events, ranging from ion channel activation to mast cell degranulation (Fig. 3). Potential cellular targets relevant to migraine are likely both in the CNS, such as the hypothalamus and TNC, and in the periphery, such as in the meninges and trigeminal ganglia, where numerous cell types express both CGRP and PACAP receptors (Fig. 2) [28, 70, 99, 182]. CGRP and PACAP actions on these cells potentially activate similar intracellular signals leading to peripheral and central sensitization. Yet despite these similarities, the differences between CGRP and PACAP actions in people and rodents suggest divergent intracellular pathways and targets. However, before we can better understand these differences, a key step will be to identify the relevant receptors for each peptide.

For CGRP, there are two receptors with approximately equal affinities [26]. The canonical CGRP receptor is a heterodimer of the GPCR calcitonin receptor-like receptor (CLR) and receptor activity-modifying protein 1 (RAMP1). A second CGRP receptor, AMY_1_, is a heterodimer of the GPCR calcitonin receptor (CTR) and RAMP1. Both can activate cAMP pathways [183] (Fig. 3). However, a direct comparison is needed between the receptors given the heterogeneity of intracellular cAMP targets seen so far with the canonical receptor [169, 184]. While the relative contributions of the two receptors in migraine remain to be established, a role for AMY_1_ is supported by the ability of AMY selective ligands to cause migraine in people [40] and light aversion, touch sensitivity, and grimace in mice [40, 185]. While CGRP has a lower affinity for the adrenomedullin receptors (CLR/RAMP2 and CLR/RAMP3), given the ability of adrenomedullin to induce migraine-like attacks similar to CGRP [46], perhaps CGRP actions via these receptors should not be ignored.

For PACAP, the canonical receptors are the GPCRs VPAC_1_, VPAC_2_, and PAC_1_ [186], which all activate adenylate cyclase and increase intracellular cAMP levels analogous to both CGRP receptors [187, 188] (Fig. 3). However, one difference between PACAP and CGRP receptors may be the ability of PACAP receptors to recruit a noncanonical cAMP signaling pathway involving the Exchange Proteins directly Activated by cAMP (EPACs) [189, 190]. The EPACs are cAMP-activated guanine nucleotide exchange factors that activate small GTPases and thus expand the diversity of cAMP signaling pathways beyond the long-recognized canonical pathway involving protein kinase A [191]. Among these EPAC targets are MAP kinases [192], although PKA and endosomal β-arrestin complexes can also activate MAP kinases [193]. Whether CGRP receptors also use EPACs is not as well established. CGRP may recruit EPACs in macrophages [194], but in dendritic cells it seemingly does not [195]. In a study with primary cardiovascular cells, activation of ERK1/2 MAP kinase by CGRP acting at the adrenomedullin receptor (a low affinity member of the CGRP receptor family) was shown to be mediated by a Gi/o pathway, while adrenomedullin used a combination of Gq/11/14 signaling and EPAC activation not used by CGRP to activate ERK1/2 MAP kinase [196]. This example of biased agonism illustrates the diversity of different G protein couplings and their downstream signaling pathways for a receptor closely related to the canonical CGRP receptor. Interestingly, there is a long established connection between EPAC signaling and pain [197], although most evidence to date places EPAC signaling upstream of CGRP, leading to CGRP release from nociceptive neurons [191]. Thus, while both PACAP and CGRP signal via cAMP, there is the possibility that they may use different cAMP signaling pathways.

An intriguing difference between the two CGRP receptors is that they have distinct internalization kinetics from the plasma membrane. Cell culture data clearly show that CGRP binding to the canonical receptor causes β-arrestin complexes and internalization to endosomes, but not the AMY_1_ receptor [198–200]. Thus, the AMY_1_ receptor potentially has prolonged cell surface signaling, while the internalized canonical receptor could continue to signal from endosomes. Importantly, endosomal signaling has been reported to be responsible for CGRP-mediated nociception [201, 202]. Likewise, endosomal signaling has been reported from the PAC_1_ receptor [193] and VPAC_1_ and VPAC_2_ can also be internalized as β-arrestin complexes in endosomes [203]. As with the two CGRP receptors, the relative contributions of cell surface and internal signaling in migraine by the multiple PACAP receptors remains an open question.

A final consideration is that until recently, the dogma was that PACAP must be acting via PAC_1_ and not the VPAC_1_ or VPAC_2_ receptors, which bind both PACAP and VIP. The rationale was primarily based on a report that VIP could not induce migraine in patients [204]. Consequently, the first PACAP-based monoclonal antibody to be tested was an antagonist to the PAC_1_ receptor. Since the trial failed to meet primary and secondary endpoints [205], this suggests either poor target engagement or possibly involvement of PAC_1_ splice variants [181], or other receptors. Indeed, VPAC_1_ and VPAC_2_ should be considered as therapeutic targets since more recent studies have shown that prolonged VIP infusion can cause delayed headache in people [60] and that VIP can induce light aversive behavior in mice if measured immediately after administration [148]. Alternatively, it is possible that PACAP involvement in migraine may be independent of VPAC_1_, VPAC_2_ or PAC_1_ receptors. PACAP can act in the trigeminal nucleus via an unidentified mechanism [107] and MrgB3 can mediate PACAP actions on mast cells in rats [156]. Another less characterized candidate PACAP receptor may be GPR55 [206]. Hence, there are no shortage of candidate receptors for PACAP actions relevant to migraine, any of which has the potential for different intracellular signaling pathways from CGRP.

## Conclusion

CGRP plays an integral role in migraine. However, CGRP alone cannot account for all cases of migraine. The neuropeptide PACAP is likely to play a related, but distinct role as CGRP based on similarities and differences observed in both clinical and preclinical studies. The PACAP pathway appears to be independent of the CGRP pathway in rodent models [144, 163] suggesting that CGRP and PACAP act by parallel paths that converge downstream of their receptors. The existence of multiple CGRP and PACAP receptors provides a plethora of potential diversity in signaling pathways for each peptide. Thus, we suggest that PACAP and its receptors provide ideal therapeutic targets to complement and augment the current CGRP-based migraine therapeutics.

## Data Availability

Not applicable.

## Abbreviations

AMY_1_: Amylin1 receptor
CGRP: Calcitonin gene-related peptide
CLR: Calcitonin receptor-like receptor
CTR: Calcitonin receptor
CNS: Central nervous system
EPAC: Exchange Proteins directly Activated by cAMP
GPCR: G protein-coupled receptor
GTN: Glyceryl trinitrate
IP_3_: Inositol 1,4,5-triphosphate
K_ATP_: ATP-sensitive potassium channel
MCA: Middle cerebral artery
MMA: Middle meningeal artery
Mrg: Mas-related GPCR
PACAP: Pituitary adenylate cyclase-activating polypeptide
PAC_1_: PACAP1 receptor
RAMP: Receptor activity-modifying protein
TNC: Trigeminal nucleus caudalis
VIP: Vasoactive intestinal peptide
VPAC: VIP-PACAP receptor
